# Three Novel ACE Inhibitory Peptides Isolated From *Ginkgo biloba* Seeds: Purification, Inhibitory Kinetic and Mechanism

**DOI:** 10.3389/fphar.2018.01579

**Published:** 2019-01-15

**Authors:** Fei-Fei Ma, Hao Wang, Chao-Kun Wei, Kiran Thakur, Zhao-Jun Wei, Li Jiang

**Affiliations:** ^1^School of Food Science and Engineering, Hefei University of Technology, Hefei, China; ^2^Anhui Habopharmqnceutical Co., Ltd., Taihe, China; ^3^Anhui Province Key Laboratory of Functional Compound Seasoning, Anhui Qiangwang Seasoning Food Co., Ltd., Jieshou, China

**Keywords:** *Ginkgo biloba*, hydrolysis, ACE inhibitory activity, purification and identification, molecular docking

## Abstract

Alcalase, dispase, trypsin, and flavourzyme were used to hydrolyze the extracted *Ginkgo biloba* seeds protein isolate (GPI). The *Ginkgo* protein hydrolyzates (GPHs) with the maximum degree of hydrolysis (DH) and ACE inhibitory activity were selected, and ultra-filtered to obtain components with different molecular weights (MW) (<1 kDa, 1–3, 3–5, and 5–10 kDa). The components with MW of <1 kDa showed better ACE inhibition (IC_50_:0.2227 mg/mL). Purification and identification by Sephadex G-15 gel chromatography and LC-MS/MS conferred three new potential ACE inhibitory peptides [TNLDWY (non-competitive suppression mode), IC_50_: 1.932 mM; RADFY (competitive inhibition modes), IC_50_:1.35 mM; RVFDGAV (competitive inhibition modes), IC_50_:1.006 mM]. Molecular docking depicting the inhibitory mechanism for ACE inhibitory peptides indicated that the peptides bound well to ACE and interacted with amino acid residues at the ACE active site.

## Introduction

Biologically active peptides are the general term for different peptides which constitute different compositions and arrangements of natural amino acids. They are known to possess a variety of biological functions such as antioxidant, immune-promoting, hormone-modulating, antibacterial, antithrombotic, antiviral, and antihypertensive activities due to multifunctional compounds derived from animal or plant proteins (Wang et al., 2017). Moreover, they have high food safety and high bioavailability which make them potential candidates for the development of functional peptide drugs and functional food additives (Sarmadia and Ismaila, 2010).

As per the World Health Organization estimate, CVD accounts for the mortality of more than 17.5 million people every year. Among the important characteristics of CVD, hypertension is an essential target for the prevention and treatment of CVD (Celermajer et al., 2012). The currently used blood pressure lowering drugs such as captopril and enalapril are limited in their use due to adverse reactions such as cough, rash, and headache (Yu et al., 2018). However, the ever-increasing incidences of CVD require some novel and safe alternatives such as food-derived active peptides. Lately, significant progress has been made in the identification and the screening of food components which positively affect the cardiovascular health (Martinez-Maqueda et al., 2012; Liu et al., 2018). Among such compounds, peptides have gained special attention due to their anti-hypertensive effect. Previously, *in vitro* hypotensive activity of peptides has been primarily determined by measuring the inhibitory activity of angiotensin converting enzyme (ACE) which is a dipeptidyl carboxypeptidase located on the cell membrane and can cause high damage by disrupting the balance between the two hormonal systems which regulate blood pressure and fluid balance (Clayton et al., 2015; Gebre et al., 2018). In addition, food-derived anti-hypertensive peptides were confirmed to exert a hypotensive effect on hypertensive patients without any toxicity or side effects (Martinez-Maqueda et al., 2012). The separation and purification of anti-hypertensive peptides from food proteins would provide a new direction for the development of natural anti-hypertensive drugs.

*Ginkgo biloba* seeds resources have been widely studied across the world due to their remarkable biological activity and pharmacological action. *Ginkgo* seed, as a Traditional Chinese Medicine, has a history of more than 600 years, but little is known about its main active components. Only few studies reported the antioxidant, anti-fatigue, and bacteriostatic effects of *G. biloba* seeds protein isolate (Lena and Philip, 2002; Huang et al., 2010). Wu et al. (2013) hydrolyzed *Ginkgo* peptide with alcalase and pepsin to obtain two antioxidant peptides with an ability to scavenge free radicals and inhibit the lipid peroxidation. However, *Ginkgo* hypotensive peptides need to be explored further to unravel their underlying mechanisms for higher action.

Herein, we used four kinds of proteases to hydrolyze GPI, in order to choose one of the hydrolyzates with high ACE inhibitory activity and perform ultra-filtration to obtain components with different MWs. The effects of MWs on ACE inhibitory activity of GPHs were studied. Also, the relationship between amino acid composition and peptide activity were established. The newly extracted ACE inhibitory peptides were purified, identified, and synthesized; their IC_50_ values were tested and peptide inhibition patterns by Lineweaver-Burk plots were explored. We also elucidated the ACE inhibition mechanism using molecular docking analysis.

## Materials and Methods

### Materials

The *G. biloba* L. seeds were purchased from Linyi city of Shandong Province, China. The seeds were shelled, husked and used for further experimentation. Angiotensin I- converting enzyme and hippuryl-l-histidyl-L-leucine (HHL), Hippuric acid were purchased from Sigma (St. Louis, MO, United States) and Yuanye Bio-Technology (Shanghai, China), respectively. Captopril was procured from MedChem Express (NJ, United States).

### Preparation of GPI

The *G. biloba* L. seeds were dried at 45°C, then crushed into powder and sieved through 80 mesh. The powder was freeze-dried after dephenolation and degreasing treatment. The resulting *G. biloba* seeds powder was dissolved in DW (1:20, w/v; pH 10.0). The above suspension was stirred and extracted for 12 h followed by centrifugation at 10,000 *g* for 30 min. The obtained supernatant was set to the isoelectric point of ginkgo protein pH 4.62 and incubated for 60 min followed by further centrifugation at 10,000 *g* for 30 min. The obtained precipitate was resuspended using distilled water, and the solution was adjusted to pH 7. Subsequently, the obtained GPI were freeze-dried until next use.

### Preparation of *Ginkgo* Protein Hydrolyzates (GPHs)

For this, 4% GPI solution was prepared and hydrolyzed separately with four proteases at their optimal hydrolysis conditions for 5 h. The enzyme dosages were 2,000 U. After hydrolysis, enzymes were inactivated at 90°C for 10 min and the solution was centrifuged at 3,000 *g* for 30 min to obtain the individual supernatants obtained from four enzymes.

### Degree of Hydrolysis

The degree of hydrolysis (DH) was calculated by referring to the method of Kimatu et al. (2017) as follow:

$$\text{DH }(\%)=\frac{\text{h}}{{\text{h}}_{\text{tot}}}\times 100=\frac{\text{B}\times {\text{N}}_{\text{b}}}{{\text{M}}_{\text{p}}}\times \frac{1}{\alpha }\times \frac{1}{{\text{h}}_{\text{tot}}}\times 100$$

where, B represented the amount (mL) of NaOH added to keep the pH constant during the hydrolysis process; N_b_ was the molar concentration of NaOH solution; M_P_ was the mass (g) of protein; α designated the average degree of dissociation in protein substrates during hydrolysis; h_tot_ was the total number of peptide bonds in the protein.

### Isolation and Purification of ACE Inhibitory Peptide

Sample ultrafiltration was carried out by using MSC300 cup type ultrafilter (MoSu, Shanghai, China). The sample was added from the feed port and the nitrogen bottle was connected to the hose fitting of the ultrafiltration cup. Then after, the ultrafiltered cup was placed and packed on the magnetic stirrer connected with the nitrogen gas bottle with a pressure not more than 0.22 MPa. Samples were sequentially passed through 1, 3, 5, and 10 kDa ultrafiltration membranes and four components were obtained (<1, 1–3, 3–5, and 5–10 kDa). The four fractions were collected, freeze-dried, and then used to test the ACE inhibitory activity. The components with higher ACE activity were selected for further purification as per the procedure given by Zheng et al. (2017). The freeze-dried hydrolyzate (200 mg) obtained after ultrafiltration was resuspended in purified water to obtain a final concentration of 50 mg/mL and subjected to further purification using Sephadex G-15 gel filtration column (1.5 cm × 60 cm). The samples filtered with a microfiltration membrane and elution were performed with distilled water at a flow rate of 1 mL/min. The sample was monitored and separated at a wavelength of 220 nm using P270 semi-preparative HPLC (Aixin, Guangzhou, China). Fractions (7.5 mL each) were collected and freeze-dried.

### Determination of ACE Inhibitory Activity

This test was performed as per the descriptions are given by the previous report by O’Loughlin et al. (2014) with some modifications. Briefly, the substrate hippuryl-histidyl-leucine (HHL, 5 mM) and samples were mixed in 0.1 M Na_2_[B_4_O_5_(OH)_4_] (pH 8.3) with 0.3 M sodium chloride. Then after, 50 μL of sample and 150 μL of the substrate were added to a centrifuge tube, mixed and allowed to stand in water at 37°C for 5 min. Subsequently, the ACE solution (50 mU/mL) was added and incubated at 37°C for 45 min. The reaction was terminated by adding 250 μL of 1M HCl and the solution was filtered through 0.45 μM nylon syringe filter before being analyzed by RP-HPLC (Waters, Milford, MA, United States) on an Inertsil ODS-3 (250 × 4.6 mm, 5 μm particle size). The mobile phase comprised of distilled water: acetonitrile = 75:25 (V/V, with 0.1% TFA) with a flow rate of 0.5 mL/min and detection at 228 nm. Captopril was used as a control and the inhibitory activity (%) was determined as below:

ACE inhibitory activity (%)=[(A−B)/A]×100

where A was the peak area of hippuric acid with the sample, B was without the sample.

### LC-MS/MS Analysis and Identification of Purified Peptide Sequences

The purified samples were analyzed by Q Exactive (Thermo Fisher Scientific, MA, United States) using an Easy-nLC 1000 (Thermo Fisher Scientific, MA, United States). The sample was desalted and lyophilized, reconstituted in 0.1% FA solution and stored at −20°C until next use. The two mobile phases were as follow: mobile phase A constituted an aqueous solution of 0.1% formic acid, and mobile phase B was a 0.1% formic acid aqueous solution of acetonitrile (acetonitrile was 84%). After equilibration of the column with 95% of the A solution, the sample was loaded from the autosampler to the Trap column. The mass-to-charge ratio of fragments of peptides was collected as follow: total 20 fragment patterns were acquired after each full scan. The mass spectrometry test raw file was searched using Mascot 2.2 software for the corresponding database.

### Synthesis of Peptides

Potential ACE inhibitor peptides identified by LC-MS/MS were synthesized at GL Biochem (Shanghai, China). The purity of the synthetic peptides was further confirmed by HPLC which was greater than 95%.

### Kinetics of ACE Inhibition

The kinetic inhibition model of *G. biloba* peptide was tested using the method of Lin et al. (2017). Briefly, different concentrations of substrate HHL (0.5, 1, 2, and 5 mM) were allowed to react with ACE. The Lineweaver-Burk plot was based on the reciprocal of the reaction rate (1/v) and the substrate concentration (1/[s]). The intercepts on the X- and Y-axes represented the reciprocals of Km and Vmax, respectively.

### Docking Analysis

For this, three-dimensional structure file of ACE protein (PDB ID: 1O8A) from RCSB Protein Data Bank (PDB^1^) was downloaded. The three-dimensional structure of *G. biloba* peptides was drawn using the molecular simulation software Discovery Studio 3.5. The hydro processing was performed and the energy was minimized by the CHARMM program. In addition, Zn^2+^ was retained in the ACE model. The DS 3.5 software scored the docking result according to its own scoring function, based on the scores and the combined free energy of each result. Among all the results, a better matching result was selected.

### Statistical Analysis

One-way ANOVA, followed by Duncan’s multiple-range tests using SPSS Statistics 20.0 (SPSS, Inc., Chicago, IL, United States) was used for data analysis with a significance difference (*P* ≤ 0.05).

## Results

### DH and ACE Inhibitory Activity of GPHs

**FIGURE 1:**
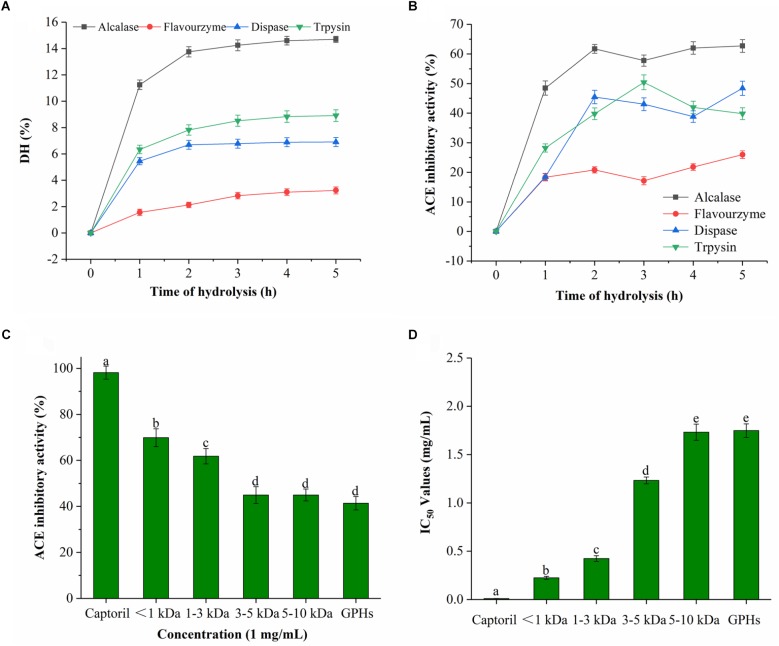
DH and ACE inhibitory activity of GPHs. **(A)** Degree of hydrolysis (DH %) of GPI hydrolyzed by alcalase, dispase, trypsin, and flavourzyme. Values are presented as means ± SD (*n* = 3). **(B)** Time course of ACE inhibitory activity of hydrolyzates generated by different proteases. Sample concentration (2 mg/mL). **(C)** ACE inhibitory activity of GPHs and membrane fractions. Sample concentration (1 mg/mL). **(D)** IC_50_ values of GPHs and membrane fractions. Captopril as a positive control. Mean ± SD (*n* = 3); different letters in the same line indicate significant differences (*p* < 0.05).

As it can be seen from Figure 1A, the DH was prolonged with time during the entire hydrolysis process. DH increased rapidly prior to 2 h interval, then after, the rate of increase gradually slowed down with time. In general, the rate of hydrolysis was high for initial 1 h, which was reduced or became constant afterward. Among the four proteases, Alcalase had the highest DH which reached 14.7% after 5 h, followed by trypsin (8.91%) and dispase (6.91%). The lowest DH was observed for flavourzyme, which was only 3.23% at 5 h. These results implied that GPI was cleaved by proteases at different sites which resulted in protein hydrolyzates with different peptide compositions.

As for the ACE inhibitory activity of the four protease hydrolyzates, the activity was increased with DH over the period of time. As shown in Figure 1B, the inhibitory activities of alcalase, trypsin, dispase, and flavourzyme at 5 h were 62.70, 39.81, 48.39, and 25.95%, respectively. Due to their different cleavage sites, different peptides with diverse ACE inhibitory activities were obtained. Among them, the activity of the alkaline hydrolyzate was relatively stronger which indicated that alkaline protease can effectively produce a hydrolyzate with higher ACE inhibitory effect.

### ACE Inhibitory Activity of GPHs and Membrane Fraction

The ACE inhibitory activity of the GPHs and the resulting fractions was significantly dependent on MW as shown in Figures 1C,D. At 1 mg/mL sample concentrations, the inhibitory activity of ACE gradually increased as the MW of the components was decreased. The inhibition activity at 3–5 and 5–10 kDa were 44.94% (IC_50 =_ 1.257 mg/mL), 44.04% (IC_50_ = 1.765 mg/mL), respectively. Subsequently, activity gradually increased with the lower MW and reached the highest level (69.86%; IC_50_ = 0.224 mg/mL) at <1 kDa.

### Purification of *Ginkgo* Peptides

**FIGURE 2:**
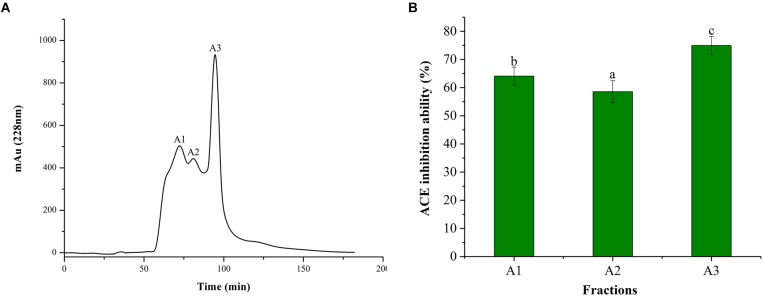
Chromatograms and ACE inhibitory activity. **(A)** Purification from the fraction of <1 kDa by Sephadex G-15 chromatography. **(B)** ACE inhibitory activity of each fraction. Different letters in the same line indicate significant differences (*p* < 0.05).

Alcalase is often used for the preparation of active peptides due to its broad specificity and strong ability to degrade proteins. From the ultrafiltration treatment, it was revealed that the ACE inhibitory activity improved with the decreasing MW of the polypeptide. Therefore, the peptide of the <1 kDa fraction was further purified using Sephadex G-15 column. It can be seen from Figure 2A, Sephadex G-15 was divided into three components (A1, A2, and A3). The three components were collected and lyophilized, and their ACE inhibitory activities were measured. The results are shown in Figure 2B. The ACE inhibitory activities of the three components (1 mg/mL) were 64.08, 58.55, and 74.96%, respectively. Therefore, the most active A3 component was selected for structural identification of the next step.

### Identification of *Ginkgo* Peptides and Peptide Synthesis

**FIGURE 3:**
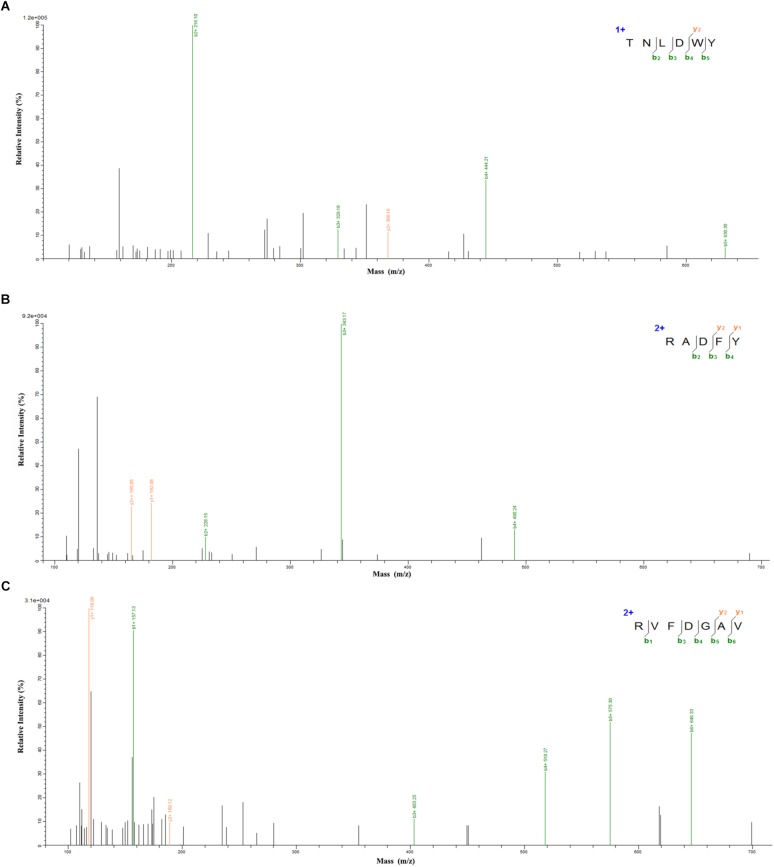
Mass spectra of peptides identified by LC-MS/MS. **(A)** TNLDWY, **(B)** RADFY, **(C)** RVFDGAV.

**FIGURE 4:**
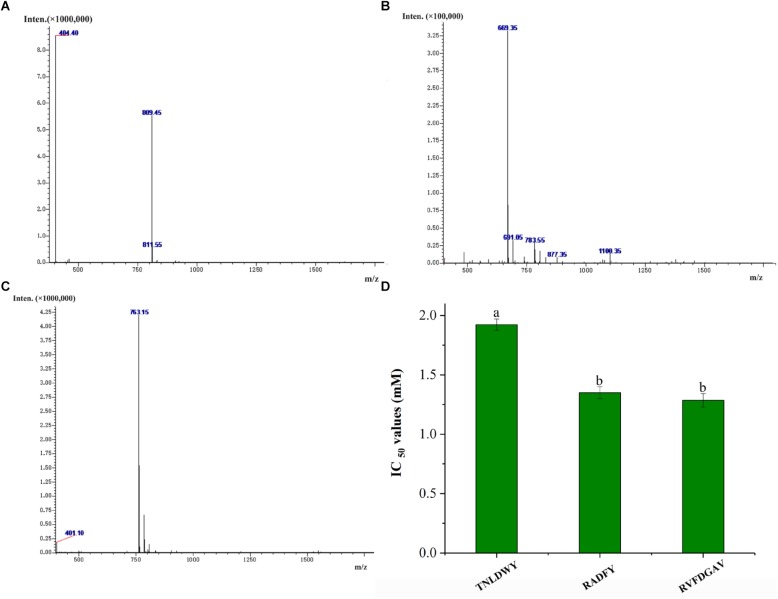
Mass spectra of synthetic peptides and IC_50_ values of synthetic peptides. **(A)** TNLDWY, **(B)** RADFY, **(C)** RVFDGAV, **(D)** IC_50_ values of synthetic peptides. Values are presented as means ± SD (*n* = 3). Different letters in the same line indicate significant differences (*p* < 0.05).

In order to identify the potential ACE inhibitory peptide, the most active component A3 was analyzed by LC-MS/MS (Supplementary Table S1). Based on this, three peptides were screened from the A3 component and their amino acid sequences were Thr-Asn-Leu-Asp-Trp-Tyr (TNLDWY), Arg-Ala-Asp-Phe-Tyr (RADFY), and Arg-Val-Phe-Asp-Gly-Ala-Val (RVFDGAV) (Figure 3). The identified sequences of these peptides were composed of 5–7 amino acid residues. To identify the ACE inhibitory activity of these three peptide fractions, the peptides were chemically synthesized. The obtained synthesized peptides were identified by mass spectrometry (Figures 4A–C). The results showed that the IC_50_ values of TNLDWY, RADFY, and RVFDGAV were 1.932, 1.35, and 1.006 mM, respectively (Figure 4D).

### Inhibitory Mechanism of *Ginkgo* Peptides

**FIGURE 5:**
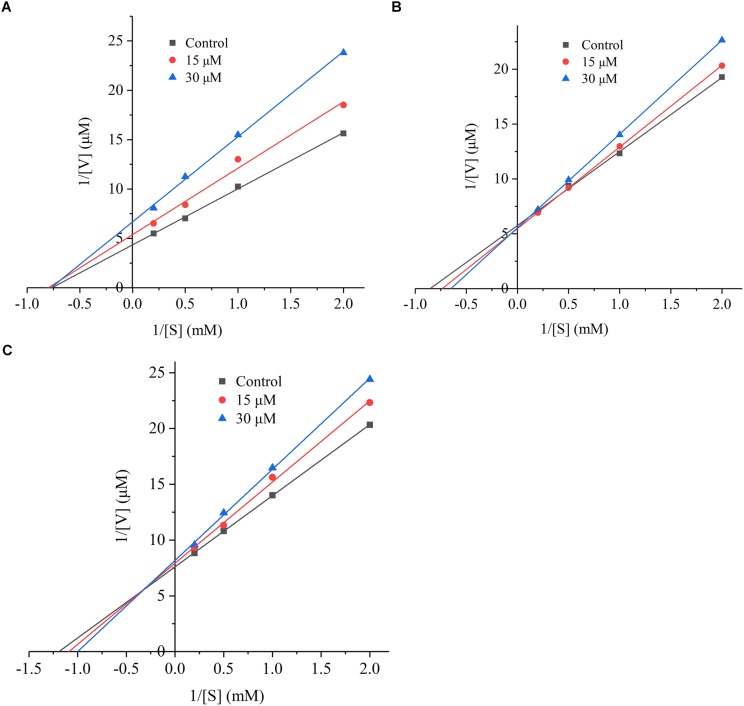
The Lineweaver-Burk plot of the inhibitory effects on ACE activities of peptides. **(A)** TNLDWY, **(B)** RADFY, and **(C)** RVFDGAV. The ACE activities were determined in the absence and presence of different concentrations of the peptides (0, 15, and 30 μM).

The inhibition mode of *G. biloba* peptide was analyzed according to the Lineweaver-Burk plot. As it can be seen from Figure 5, when *Ginkgo* peptide TNLDWY was added, both Vmax and Km were changed, Vmax increased with the increasing peptide concentration; whereas, Km did not change significantly with peptide concentration which indicated that its inhibition pattern may be a non-competitive inhibition mode. For RADFY and RVFDGAV, Vmax did not change significantly with concentration; while, Km increased with the increasing peptide concentration, indicating that both peptides are competitive inhibitors, which can bind to the active sites of ACE, thus blocking the binding of ACE to the substrate and inhibiting the activity of ACE.

### Molecular Docking Simulation Between Peptides and ACE

**FIGURE 6:**
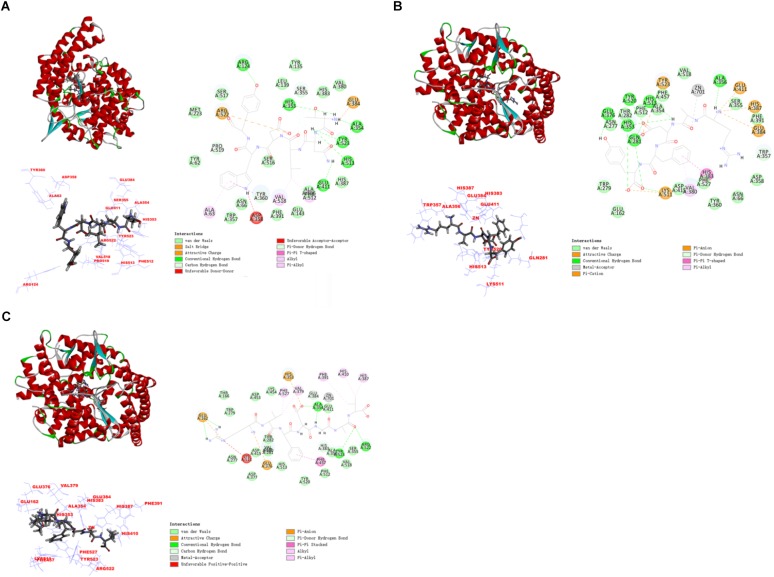
The molecular docking simulations of TNLDWY, RADFY, and RVFDGAV with ACE (PDB: 1O8A). **(A)** General overview, local overview, and 2D-diagram of docking pose of peptide TNLDWY; **(B)** general overview, local overview, and 2D-diagram of docking pose of peptide RADFY; **(C)** general overview, local overview, and 2D-diagram of docking pose of peptide RVFDGAV.

In this study, the interaction of three peptides with ACE was evaluated. The results showed that all the peptides could bind well to ACE and form a stable complex, indicating their use as a potential ACE inhibitor. The score of – Cdocker Interaction Energy was shown in Table 1. The scores of TNLDWY, RADFY, and RVFDGAV fractions were 102.995, 105.335, and 117.706, respectively. Molecular docking results were shown in Figure 6 and Table 2. The optimal docking position of TNLDWY can form six hydrogen bonds, among them it formed hydrogen bonds with Ala 354, Tyr523 in S_1_ active pocket, His353, His513 in S_2_ active pocket, and Zn^2+^ residues and it was found stably bound with all of them. This may be related to the strong ACE inhibitory activity of peptide. It was observed from the Figure 6B that RADFY can form seven hydrogen bonds with amino acid residues, and intermolecular hydrogen bonds with the residues Gln281, His353, His513, and Tyr520 in the S_2_ active pocket. The formation of these hydrogen bonds greatly stabilized the enzyme-peptide complex. In addition, RADFY formed an ionic bond with Zn^2+^, conjugate forces with Val380, His383, Glu384, His387, Glu411, Lys511, and Tyr523 as well as van der Waals force with amino acid residue Trp356. These interactions may result in the deformation of the Zn^2+^ ligand and the inactivation of ACE. Compared to TNLDWY and RADFY, RVFDGAV can form four hydrogen bonds with amino acid residues, which was stably connected to ALA354, TYR523 in the S_1_ active pocket, and Glu162 in the S_1_′ active pocket. However, RVFDGAV could form ionic bonds with Zn^2+^, which directly led to the deactivation of ACE molecules. In addition, it formed a conjugate with the amino acid residues such as Glu162, His353, Glu376, Val379, His387, Phe391, His410, Phe457, and Phe527 (Figure 6C).

**Table 1:** Computational modeling energy scores and interaction results of the top ranked poses of docked peptides and ACE (PDB: 1O8A).

Peptide sequence	-Cdocker interaction energy	Number of predicted hydrogen bonds	Number of ACE residues
TNLDWY	102.995	6	6
RADFY	105.335	7	10
RVFDGAV	117.706	4	5

**Table 2:** ACE residues in coordination with Zn^2+^ and amino acids of S1, S2 and S1′ active site involved in interaction with selected peptides after molecular docking simulation.

ACE residue	TNLDWY	RADFY	RVFDGAV
His 383	–	✓	–
His 387	–	✓	✓
Glu 411	✓	✓	–
Ala 354	✓	–	✓
Tyr 523	✓	✓	✓
Glu 384	✓	✓	–
Gln 281	–	✓	–
His 353	✓	✓	✓
Lys 511	–	✓	–
His 513	✓	✓	–
Tyr 520	–	✓	–
Glu 162	–	–	✓

## Discussion

In recent years, food- derived ACE inhibitory peptides from plant protein have attracted more and more attention due to their fewer side effects. In this study, alcalase, dispase, trypsin, and flavourzyme were used to hydrolyze *Ginkgo* protein. The DH and ACE inhibitory activity of the *Ginkgo* protein hydrolyzate obtained by hydrolysis of alcalase were reported as the highest. The hydrolysis process took place rapidly during the initial stage which resulted in hydrolysis of many peptide bonds, then after, the rate of hydrolysis was slowed down due to the gradual reduction of the substrates availability for hydrolysis (Ketnawa et al., 2017; Hu et al., 2018; Wei et al., 2018). These findings were consistent with the previous report of rapeseed protein hydrolyzates with effective ACE inhibitory action when hydrolyzed with alcalase (He et al., 2013).

Angiotensin converting enzyme is a multifunctional extracellular dipeptidase present in different tissues. The inhibitory activity of ACE lowers the blood pressure by reducing the production of angiotensin II and reducing the destruction of kinins (Zheng et al., 2017). The ACE inhibitory activity of the hydrolyzate was related to the molecular weight distribution. The ultrafiltration was used to separate the *Ginkgo* hydrolyzate into five components. At molecular weight <1 kDa, the ACE inhibitory activity of *Ginkgo* hydrolyzate was reported the highest. This observation was in accordance with previous studies reporting the higher ACE activity of low MW polypeptides (Wang et al., 2015; Ola et al., 2017). Moreover, Silvestre et al. (2012) confirmed that the ultrafiltration treatment may retain peptides (AAA) at the C-terminal position to promote the ACE inhibitory activity.

In order to further purify the highly active ACE inhibitory peptide, the *Ginkgo* component (<1 kDa) was separated by gel filtration chromatography, and the amino acid sequence was identified using LC-MS/MS. Thus, three new ACE inhibitory peptides were obtained: TNLDWY (1.932 mM), RADFY (1.35 mM), and RVFDGAV (1.006 mM) which showed strong ACE inhibitory activity. According to report by Hernández-Ledesma et al. (2011), most of the ACE inhibiting peptides were short sequences consisting of 2–12 amino acids. In addition, the activity of the ACE-inhibiting peptide was strongly related to the presence of its C-terminal amino acid, aromatic amino acid, and hydrophobic amino acid. For instance, the presence of the aromatic amino acid (Tyr, Phe, and Trp) significantly enhanced the activity of the ACE inhibitory peptide. In addition, Arg is also known to play an important role in the inhibitory peptide. Toopcham et al. (2017) showed that the inhibitory activity of the polypeptide was significantly reduced after removal of Arg. Furthermore, Liu et al. (2018) proved that amino acid such as Leu in the peptide significantly affected the ACE inhibitory activity regardless of its position at the C-terminus or the N-terminus. Similar results were also reported by Lee and Hur (2017). These studies reported the KPLL, VLAQYK, and DLP peptides from different proteins materials with an appreciable ACE inhibitory activity in the presence of hydrophobic amino acid (Leu). In our study, Tyr was located at the C-terminus of TNLDWY and RADFY. In addition, the two peptides from RADFY and RVFDGAV contained Arg at the N-terminus, and the content of hydrophobic amino acids in the three peptides was higher. Above all, the peptide fragments corresponded with the structural characteristics of ACE-inhibiting peptides.

The mode of inhibition of ACE-inhibiting peptides was observed generally non-competitive, competitive, and mixed. For short peptides (2–12 amino acids), most of them were competitive inhibitors and they attached to the ACE enzyme in order to prevent the binding of the substrate HHL. A similar study by Lin et al. (2017) proved that the peptides KYIPIQ and LPLPLL from *Qula* casein exhibited a competitive inhibition mode. However, for non-competitive inhibitors, they bind to sites other than the substrate binding site of the enzyme and ultimately affect the binding of the substrate to the enzyme. Similar patterns were observed in case of food-borne inhibitory peptides such as PFPGPIPN from *Qula* casein, and the hazelnut peptide YLVR (Lin et al., 2017; Liu et al., 2018). In this competitive mode, ACE, substrate, and peptide formed an enzyme-substrate-inhibitor complex, which prevented further release of the product and resulted in a decrease in Vmax (Barbana and Boye, 2011).

Studying the molecular interaction mechanism between ACE and inhibitory peptides is helpful for screening and designing new ACE inhibitory peptides. However, inadequate information is available on molecular interactions of inhibitory peptides. Molecular docking is based on the “lock and key principle” of ligands and receptors, simulating the interaction between small molecule ligands and receptor biomacromolecules. The mode of binding and affinity between them allows the virtual screening of drugs. ACE is a Zn^2+^-dependent carboxydipeptide enzyme, in which Zn^2+^ is an important part of the ACE active center (Pina and Roque, 2009). According to previously reported data (Pan et al., 2012), the main interaction residues on the active site of ACE were divided into three active pockets (S_1_, S_2,_ and S_1_′). S_1_ (Ala354, Glu384 and Tyr523); S_2_ (Gln281, His353, Lys511, His513 and Tyr520); and S_1_′ (Glu162 residue). At the active site of ACE, two histidines (His383 and His387) together with Glu 411 constituted a Zn^2+^ ligand. It has been reported that the inhibition of peptide-induced ACE activity may be achieved by the combination of hydrogen bond-stable enzyme-peptide complexes (Fu et al., 2017). In addition, the formation of van der Waals forces with amino acid residues such as His353, Ala 354, Ser355, Glu384, His513, and Pro519, conjugate interaction with Tyr360 and non-covalent interactions with Glu384, Arg522 may also contribute to the stability of enzyme peptide complexes. The presence of these forces will make the structure of the enzyme-peptide complex more stable, more conducive to inhibition of ACE activity, which may be the reason of strong ACE inhibitory activity of TNLDWY, RADFY, and RVFDGAV.

## Conclusion

In this study, *G. biloba* seeds were used to prepare potential ACE inhibitory peptides. GPHs prepared from different proteases displayed diverse ACE inhibitory activity *in vitro*. The highest DH and *in vitro* ACE inhibitory activities were observed in GPHs prepared from alcalase. The components obtained by ultra-filtration with alcalase showed that the smaller MW peptides conferred the stronger ACE inhibitory activity. Three potential ACE inhibitory peptides (TNLDWY, RADFY, and RVFDGAV) were obtained from the peptide fraction (<1 kDa) by LC-MS/MS. RVFDGAV showed the highest ACE inhibitory activity with an IC_50_ value of 1.006 mM and appeared as a competitive inhibitor. Molecular docking results indicated that the peptides could firmly bind to ACE and further interacted with amino acid residues at the ACE active site. Our results indicated that the peptides obtained by enzymatic hydrolysis of alcalase exhibited effective ACE inhibitory activity *in vitro*, which make them potential candidates for the development of functional foods or anti-hypertensive drugs in the future.

## Author Contributions

F-FM, HW, C-KW, and KT were involved in the project design, carried out most of the experiments, and drafted the manuscript. Z-JW and LJ contributed to the experimental design, manuscript preparation, and submission. All authors participated in writing the manuscript and/or revised it critically for important intellectual content.

## Conflict of Interest Statement

HW was employed by Anhui Habopharmqnceutical Co., Ltd. Z-JW was employed by Anhui Qiangwang Seasoning Food Co., Ltd. The remaining authors declare that the research was conducted in the absence of any commercial or financial relationships that could be construed as a potential conflict of interest.

## Footnotes

1 http://www.rcsb.org/pdb/home/home.do
