# Supplementary material for: Three Novel ACE Inhibitory Peptides Isolated From Ginkgo biloba Seeds: Purification, Inhibitory Kinetic and Mechanism
Source: Front Pharmacol. 2019 Jan 15;9:1579. doi: 10.3389/fphar.2018.01579 (PMC6340938; doi:10.3389/fphar.2018.01579)
Supplement: Supplementary file 1 [file Table_1.DOCX]

Table S1: Peptide profile of A3 fraction identified by LC- MS/MS

| Number | MH+(Da) | Charge | Sequence | Length | Score | ACE activity |
| --- | --- | --- | --- | --- | --- | --- |
| 1 | 763.4097 | 2 | RVFDGAV | 7 | 36.05 | ACE inhibitor |
| 2 | 671.3147 | 2 | RADFY | 5 | 22.41 | ACE inhibitor |
| 3 | 516.3027 | 1 | DGLIV | 5 | 21.4 | - |
| 4 | 713.3616 | 1 | YFDRL | 5 | 24.55 | - |
| 5 | 832.4451 | 1 | DLLLDFP | 7 | 24.67 | - |
| 6 | 805.4124 | 1 | ETAQLML | 7 | 21.24 | - |
| 7 | 581.2777 | 1 | SSSVTT | 6 | 29.01 | - |
| 8 | 811.3621 | 1 | TNLDWY | 6 | 22.32 | ACE inhibitor |
| 9 | 780.3563 | 1 | FDALWE | 6 | 23.26 | - |
| 10 | 702.4032 | 1 | DATVALI | 7 | 29.48 | - |
| 11 | 906.4237 | 1 | ASLEQDLM | 8 | 23.98 | - |

Charge, valence state; MH+, molecular weight of peptide.
